# Backyard Proteomics: A Case Study with the Black Widow Spider

**DOI:** 10.1021/acs.jproteome.5c00342

**Published:** 2025-07-28

**Authors:** Tarsh Shah, Jackson A. Fitzpatrick, Benjamin C. Orsburn

**Affiliations:** 1 The Advanced Academics Biotechnology Program, 1500 The Johns Hopkins University School of Medicine, Baltimore, Maryland 21205, United States; 2 Proteomic ünd Genomic Sciences, Baltimore, Maryland 21214, United States; 3 Organ Pathobiology and Therapeutics Institute The University of Pittsburgh, Pittsburgh, Pennsylvania 15223, United States

**Keywords:** metaproteomics, DIA, nonmodel organism proteomics, spiders, toxins

## Abstract

Nearly all methods of mass-spectrometry-based proteomics rely on knowing the proteome of the species. In less studied organisms without annotated genomes, it can seem impossible to perform proteomic analysis. In this study, we sought to answer the question: does enough information exist to do proteomics on any organism we want? As a case study, we started with material available due to an infestation of a home with black widow spiders. Thanks to the recent publication of an annotated genome for one species of black widow spider, we were able to identify 5502 protein groups and assign putative annotations using ortholog mapping. We also demonstrate that had we not had this resource, over 2000 proteins could be identified using other available spider genome annotations, despite their unrelatedness. Moreover, regardless of the spider proteome used, proteins annotated as toxins were almost exclusively observed in the main body of the mature female black widow spider. Overall, these results provide a draft proteome map for the black widow spider and valuable data for validating machine learning models while also suggesting that the door to insightful quantitative proteomics may already be open for millions of less studied organisms. All raw and processed proteomic data are available through the ProteomeXchange repository as accession PXD051601.

## Graphical abstract

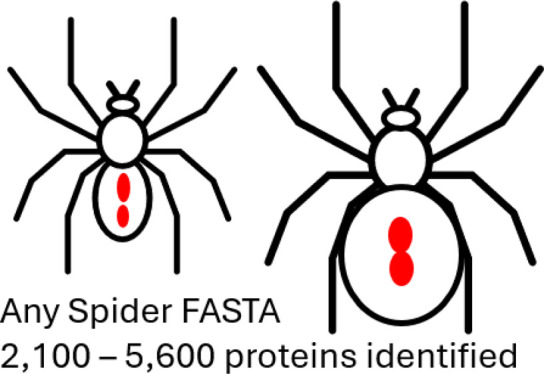

## Introduction

Quantitative proteomics can be performed using a dizzying number of technologies, many of which can employ multiple techniques. Liquid chromatography mass spectrometry (LCMS) can acquire untargeted data with either data-dependent acquisition, relying on on-board computers for on-the-fly target acquisition, or data-independent acquisition where you fragment it all and let the algorithms sort it out.^1^ One can also use LCMS systems to specifically target what you are interested in or mix and match these methods for various semitargeted methods.^2,3^ Older and newer techniques exist where proteins are targeted and measured by antibodies, through protein arrays or by oligonucleotide amplification.^4^ You can even estimate protein presence and concentration within a narrow dynamic range through aptamer binding assays.^5^ While a tipping point may be approaching soon for so-called “next generation” techniques thanks largely to their impressive throughput capabilities,^6^ most proteomics laboratories in the world probably rely on LCMS technologies at the date of this writing. While the earliest techniques in LCMS-based proteomics relied heavily on de novo sequencing tools, the productivity of the field increased exponentially in the wake of the resources produced by genomic sequencing technologies.^7^ Today, nearly every LCMS proteomic software tool relies on curated libraries of protein or peptide sequences derived from annotated high-quality genome assemblies. The form of this input is generally a FASTA file, which is a flat format of protein sequences, or spectral libraries, which are experimentally obtained spectra matched to the same FASTA.^2^ The productivity of LCMS proteomics again increased substantially due to the application of deep learning tools, which can now predict peptide fragmentation patterns from the curated knowledge of the past.^8,9^ While these deep learning tools are often described as “library-free”, this is somewhat of a misnomer as protein sequence input is still required from which peptide masses and fragmentation patterns are modeled and predicted.

The end result of these advancements is that LCMS-based proteomics has nearly universal methods that can provide proteomic data on practically any sample type, but this is largely limited to organisms with annotated genome assemblies. At the end of 2024, NCBI had annotated 1286 eukaryotic species,^10^ though this does not mean all 1286 have had proteomic analysis. An estimated 8.7 million distinct species of eukaryotic life exist on earth today, meaning that these annotated eukaryotic genomes provide minimal coverage of the tree of life.^10^ Some groups, like mammals, have more annotated genomes relative to the number of species, but even then some clades have more annotated genomes than others.^11^ This paucity of unannotated species genomes is mirrored in the published proteomic data. As of February of 2025, humans comprise 47% of the roughly 40,000 mass spectrometry proteomic data sets on ProteomeXchange, followed by mouse at 17%, then a steep drop off to 2.9% as the other classic model organisms comprise the top 10 species.^12^ We will not diminish the importance of human research but emphasize the diversity of species in the world around us that lack proteomic analysis and that this presents an opportunity for researchers everywhere.

There are different reasons to perform proteomic analysis on a given species, which can include everything from developing drugs against that organism^13^ through learning how to synthesize molecules it produces,^14^ to simple curiosity of the unknown. But should one forego exploratory analysis when there is not an annotated genome assembly? Though the specific annotated genomic landscape (i.e., what is the closest related species that is annotated) of a species will vary, we suggest that proteomic analysis should be considered viable and that the data will be valuable in the future as more genomes are sequenced and annotated. In this case study, we performed proteomic analysis on southern black widow spiders (*Latrodectus mactans*). There are around 52,000 spider (Araneae) species,^15^ and only 46 have reference genomes on NCBI. Of these, only 14 have annotated genome assemblies (as of August 2024). The black widow spider is in the Theridiidae family, which has one annotated genome assembly on NCBI, *Parasteatoda tepidariorum*. While multiple proteomic studies have focused specifically on the toxins produced by black widow spiders,^16,17^ no comprehensive proteomic analysis has been performed on the whole spider. Based on the availability of specimens due to persistent infestation of a home in Pennsylvania, we have sampled two individuals: a single intact spiderling and a mature spider, with the latter in a partially homogenized state due to over-reaction of a nervous mass spectrometrist during sample collection. In total, six separate anatomical regions could be excised from these organisms for proteomic analysis. After initial data analysis, the Western black widow spider (*Latrodectus hesperus*) genome assembly was published^18^ and upon request, authors provided the FASTA for the annotated genome, albeit without useful protein names. Using this *L. hesperus* annotated genome, we provide here a draft proteome map of the black widow spider but also compare it to proteomic analysis that simulate conditions as if we only had the 14 other spider genome FASTA databases. The analysis using the *L. hesperus* annotated genome was unsurprisingly superior, but proteomic analysis using the 14 NCBI spider genome annotations provided valuable results, including putative biological insights. These results highlight that proteomic analysis of specimens from the world around us (even our homes and backyards) can be useful even when a species-specific FASTA does not exist.

## Methods

### Spider Collection

Following multiple attempts to collect protein from spiders homogenized by an aluminum baseball bat (Marucci F5, Baton Rouge, Louisiana), an author missed directly striking what appeared to be an adult female spider. This specimen was only partially homogenized and likely incapable of injuring the author, who made undignified sounds while transferring her into a 15 mL centrifugation tube (Falcon). The adult spider sex was inferred from the presence of webbed egg sacs in the general direction and from morphological details from various online sources. A second immature spiderling was later captured fully intact using an eco-friendly bug vacuum for kids (Nature Bound Toys, Denver, Colorado). The spiderling was transferred to a 15 mL centrifuge tube, and both samples were stored at −20 °C before transporting to the lab on wet ice for long-term −80 °C storage. Due to the small size of the spiderling and general inexperience of the authors, no sex was inferred for the intact spiderling. It should be noted that based on previous invertebrate studies to be published elsewhere, we had cleared with the Johns Hopkins Animal Care and Use Committee that all insects and arachnids were treated as *Drosophila melanogaster* studies and required no additional clearance for their study. However, we attempted to minimize suffering to these organisms wherever possible.

### Spider Protein Sample Preparation

The *Latrodectus* specimens were aseptically transferred to a 15 mL falcon tube for further processing. Upon thawing, the specimens were dissected by best effort into discernible sections. For the intact spiderling, four distinct regions were excised. For the adult spider for which capture was more challenging and resulted in partial homogenization, only two regions could be confidently collected and were designated as “body” and “legs”. Homogenization was carried out using a bead mill (BeadBug 3, Benchmark Scientific, Sayreville, New Jersey) in 2 mL bead chambers, employing 30 s pulses of creepy spider parts in 700 μL of 1× S-Trap lysis buffer (5% sodium dodecyl sulfate; Protifi, LLC). Following homogenization, the resultant lysates were centrifuged at 13,000*g* for 3 min to facilitate clarification, thereby separating the liquid fraction protein fraction from solid chunks of chitin.

The clarified homogenates were prepared by suspension trapping^19^ using S-Trap Mini spin columns (Protifi) with minor modifications of established manufacturer protocols. Protein samples solubilized in 1× S-Trap lysis buffer were reduced with dithiothreitol (DTT; Thermo Fisher; final concentration: 10 mmol/L) at 60 °C for 20 min followed by alkylation with iodoacetamide (IAA; Thermo Fisher; final concentration: 40 mmol/L) at room temperature in a dark drawer for 20 min. A total of 40 μL of clarified homogenate was acidified with 12% phosphoric acid and diluted 1:10 (volume fraction) in S-Trap binding buffer (80% LCMS-grade methanol with 100 mmol/L triethylammonium bicarbonate, pH 8.0) and centrifuged at 2000*g* for 2 min to bind the proteins to the S-Trap column. The bound proteins were then washed three times with 300 μL of the binding buffer. Finally, the clean bound proteins were digested by the addition of 10 μg of MS-grade trypsin (Thermo Scientific) per sample in a total of 80 μL of 100 mmol/L triethylammonium bicarbonate solution, resulting in the enzymatic cleavage of proteins into peptides. Following digestion overnight (approximately 16 h) at room temperature, peptides were eluted with 0.2% formic acid (volume fraction) in water and dried under vacuum centrifugation (Eppendorf). Subsequently, they were resuspended in 200 μL of 0.1% formic acid (volume fraction) in water, and their concentrations were quantified using the Pierce Quantitative Colorimetric Peptide Assay kit (Thermo Scientific) following the vendor protocol.

### LCMS Analysis

The peptides were diluted, and 400 ng of peptides per sample was loaded onto each individual EvoTip (EvoSep, Odense, Denmark) and injected into timsTOF FleX mass spectrometer (Bruker) through an Evosep One (Evosep, Odense, Denmark) liquid chromatography system. The 30 sample per day (SPD) separation program was used, which is a 44 min separation on a 15 cm × 150 μm C-18 column with 1.5 μm particle size (PepSep, Odense, Denmark). The outlet column was connected through a “zero dead volume” union to a CaptiveSpray emitter with a 10 μm emitter tip. Eluted peptides were analyzed with diaPASEF using a method constructed from the default “diaPASEF short gradient” in Bruker TIMSControl 4.0.

### Data Analysis

The resulting mass spectrometry (.d) data files were automatically converted by using a batch command in Bruker Hystar to HTRMs with appropriately named “HTRMS Converter” software (Biognosys). The files were then transferred to a central server for processing in SpectroNaut 19. Search settings for SpectroNaut followed the default DIA+ “deep” workflow for the timsTOF data. Briefly, SpectroNaut determined the appropriate precursor and fragment ion tolerances with carbamidomethylation of cysteines set as a static modification. Oxidation of methionine and acetylation of the protein N-terminus were the sole dynamic modifications considered, with a maximum of three dynamic modifications considered on each peptide sequence. Up to 2 missed tryptic cleavage sites were considered. For downstream analysis, a pivoted report for the proteins and peptides was separately exported using the default templates with the additions of the protein description and gene identifiers, which are strangely absent in both default templates. A protein FASTA of the recently published genome assembly of the Western black widow spider (*L. hesperus*; taxonomy ID: 256737) was obtained from the authors.^18^ This genome assembly is available on Genbank as ASM3797512v2 (GCA_037975125.2) but has not been annotated by RefSeq, which is why we used the author’s supplied FASTA. This FASTA was used for *L. hesperus* specific searching is labeled 68345-CDS-prot.fasta (34 991 sequences). The original CDS matches and linked annotations are provided as Supporting Information 1, while the other 14 spider FASTA are provided as Supporting Information 2. Note that the *L. hesperus* FASTA was provided mislabeled within FASTA headers as *Hesperus amabilis*, but this was simply a mistake in the system. A helpful peer during the review process suggested the reanalysis of these files with evolutionarily distant databases to help determine the level at which these data may be affected by random matches. Without any better ideas, the files were reanalyzed with the Uniprot Human reviewed database on hand (downloaded April 25, 2025) and the *Arabidopsis thaliana* database from the same source (downloaded June 24, 2025). Further reviewer comments led us to seek out more closely related organisms outside of spiders for comparative analysis; as we have utilized all currently annotated protein databases for Arachnids in this study, we chose the American cockroach thanks to advice from colleagues. This database was downloaded on July 11, 2025 from UniProt/SwissProt.

### Ortholog Mapping of Protein Identifications

This FASTA used had no useful information for protein descriptions in the FASTA header, and so ortholog mapping was used to assign putative identifications to allow proteomic results to be interpreted. Briefly, make_subset_DB_from_list_3.py and db_to_db_blaster.py from the PAW_BLAST GitHub repo were used locally to BLAST (v.2.11.0+) each of the identified protein entries against a FASTA composed of all the UniProt entries under the taxonomy Araneae (Spider Order; taxonomy ID: 6893) from the 2024_07 UniProt release (614 548 entries). This UniProt FASTA was used because the naming and descriptions were more complete than using any of the 14 NCBI annotated genome assemblies. We expect there to be an official NCBI annotated black widow genome assembly in the future that will have curated protein names and descriptions adhering to RefSeq levels of quality. These approximately 5500 mapped protein names were not checked for poor or no homology but instead were used for descriptive purposes and should be considered putative.

### Analysis of Toxin Proteins

All output protein reports were exported as.tsv files from SpectroNaut using the default protein pivot report option with the addition of the Protein Group Description column enabled. The.tsv files were combined into a single Excel file with 16 sheets, and each sheet was filtered in place on the term “toxin”. The resulting lists were combined into a single Excel sheet, and the RAC-1 proteins were manually deleted as this ubiquitous Eukaryotic protein description contains the letters “toxin” but is clearly unrelated. The sheet was then copied into the Broad Morpheus visualization program using default parameters for heatmap creation (https://software.broadinstitute.org/morpheus).

## Results and Discussion

### Generating a Draft Proteome Map

The majority of spider research, including specifically the black widows, is focused on silk^17,20^ and toxins,^21,22^ largely ignoring the rest of the spider. As of February 2025, ProteomeXchange lists 29 data sets of spider proteomes, with only 2 including multiple tissues, with the obvious exception of the data published with this study. Though this study was advantageous in nature, it still offered an opportunity to provide a draft proteome for the black widow spider due to multiple tissue samples. Though unavailable until well after the initial data analysis, the *L. hesperus* annotated genome allowed for peptide identifications in the DIA mass spectrometry data. The identifications in each tissue ranged from 2422 to 39,280 peptides, with the most identified in the adult body and the least in the small head of the immature spiderling. These in turn resulted in 5,608 protein identifications across the experiment, ranging from 1135 in the spiderling head to 5602 in the legs of the adult spider. Due to challenges acquiring the adult spider previously mentioned in this text, the two sections contained a homogenate of more anatomical regions than the spiderling samples. It should also be noted that the spiderling was quite small and excision of the head was difficult and resulted in limited peptides for analysis. The gene annotation itself in the *L. hesperus* annotated genome seemed accurate in the sense that it explained the collected data well enough but the gene identifications themselves were not informative. To overcome this limitation, we mapped each identified protein to its Araneae (Spider Order; taxonomy ID: 6893) ortholog in order to provide putative identifications. This complete proteome map with putative protein IDs and relative abundance across all tissues is available in Supporting Information 1. The dynamic range of each tissues proteome is plotted and the top five most abundance proteins are available in Supporting Information 4: Figures S1–S6, and the underlying data was derived from Supporting Information 1: Table S1. Finally, we have broken up the identifications by tissue (and by individual) and sorted from highest to least abundance. While it is often considered valuable to determine the most abundant proteins expressed in each respective tissue, we acknowledge that without more replicates (both biological and experimental), any subtle differences in abundance should be interpreted with caution. Overall, to our knowledge this is the first multitissue proteomic analysis of this species or genus (Figure 1).

**Figure 1:**
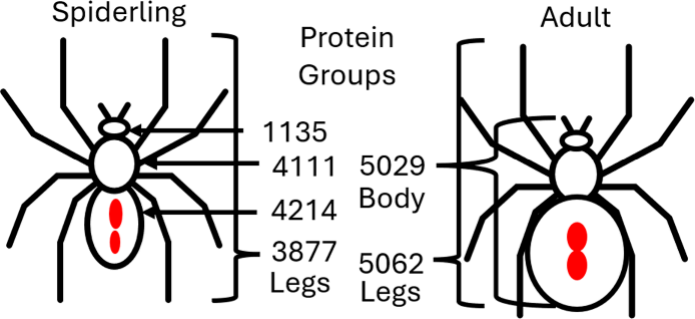
Schematic representing the samples that were taken for proteomics and the number of protein groups identified in each sample when using translated coding DNA sequences from the Western Black Widow genome.

### Comparison to Nonspecies-Specific Search Results

A major roadblock to performing proteomics in many species is the lack of a species-specific annotated genome against which to search the mass spectrometry data. During this study, we were able to use a newly annotated genome, but we wanted to examine how the results might have looked without a species-specific annotated genome. To demonstrate this we analyzed the data using each of the 14 spider genome annotations (Table 1, Supporting Information 2). Unsurprisingly, the results of using the species-specific genome annotation was better than using these other species with 50,699 experiment-wide peptide identifications. Using identified peptides as a comparison measure, we found the worst performing spider genome annotation to be *Trichonephila clavipes* with 8274 IDs, 16.3% of peptide IDs versus the species-specific genome, and the best performing spider to be *P. tepidariorum* (the common house spider) with 12,361 peptide IDs, 24.4% on peptide IDs versus the species-specific genome. Interestingly, in the case of the *P. tepidariorum* results, of the 12,361 peptide identifications, 10,151 peptides were shared with the species-specific search. These differences seem to recapitulate the relatedness of these species (both the black widow and common house spider are in the Theridiidae family), which reiterates that when using a nonspecies-specific genome annotation, it is best to use the closest relative possible.

**Table 1:** Summary Table of the Number of Peptide Groups, Proteins, and Protein Groups Obtained when Files Were Searched against Each FASTA Separately with Scientific and Common Names for Each Organism

FASTA file used	taxonomy ID #	peptides	proteins	protein groups
*Araneus ventricosus* (orb weaver)	182803	9913	3829	2464
*Argiope bruennichi* (wasp spider)	94029	10,752	3442	1956
*Trichonephila clavata* (Joro spider)	2740835	10510	2889	2344
*T. clavipes* (banana spider)	2585209	8335	2542	1968
*Trichonephila inaurata madagascariensis* (golden orb weaver)	2747483	9657	2377	1980
*Nephila pilipes* (giant golden orb weaver)	299642	10,471	2754	2156
*P. tepidariorum* (house spider)	114398	12,361	4396	2347
*Uloborus diversus* (feather-legged lace weaver)	327109	9962	2777	2028
*Stegodyphus dumicola* (African social spider)	202533	10,003	3083	1974
*Stegodyphus mimosarum* (velvet or social spider)	407821	9805	2098	2044
*Oedothorax gibbosus* (humped orb weaver)	931172	9611	2610	2045
*Caerostris darwini* (Darwin’s bark spider)	1538125	10,658	2542	2110
*Caerostris extrusa* (broad-headed bark spider)	172846	10,272	2143	1902
*Larinioides sclopetarius* (bridge spider)	280406	10,897	3786	2250
*L. hesperus* (Western black widow spider)	256737	50,699	5608	5502
*Homo sapiens* (human)	9606	1236	629	498
*A. thaliana* (Thale cress)	3702	196	182	98
*Periplaneta americana* (American cockroach)	6978	2067	1494	576

### High Relative Concentrations of Toxin Proteins Are Observed in the Mature Female Spider

Finally, we performed an exploratory analysis using the species-specific results for toxins present across tissues. In every case, each annotated FASTA file used provided a minimum of 7 protein group annotations with a description containing the world “toxin”. Following removal of RAC1 protein, which are highly conserved RAS-related botulinum toxin substrate proteins and unrelated to spider toxins, a minimum of six toxin were identified in all analyses (Supporting Information 3). When visualizing the abundance of these proteins using row-normalized heatmaps (Figure 2), we find that nearly all were primarily observed in the mature female spider body with lower relative levels in the female legs. Toxin proteins were only sporadically detected in the immature spiderling. Recent focused work on *Latrodectus* toxins described the bite of a mature spider as an “arsenal” of various toxins with specificity to a broad range of organisms. Some of these proteins appear to affect only mammals, while others have broad impacts across all vertebrates. Immature spiderlings do possess toxins, and extracts from immature spiderlings have demonstrated toxicity in some animal testing. It is thought, however, that while spiderlings have toxins for insects for paralyzing and consuming them, the full arsenal of toxins are reserved for mature females when protecting their eggs and young.^23^ The proteomic data appear to support this hypothesis, regardless of the FASTA database utilized in the analysis of these files.

**Figure 2:**
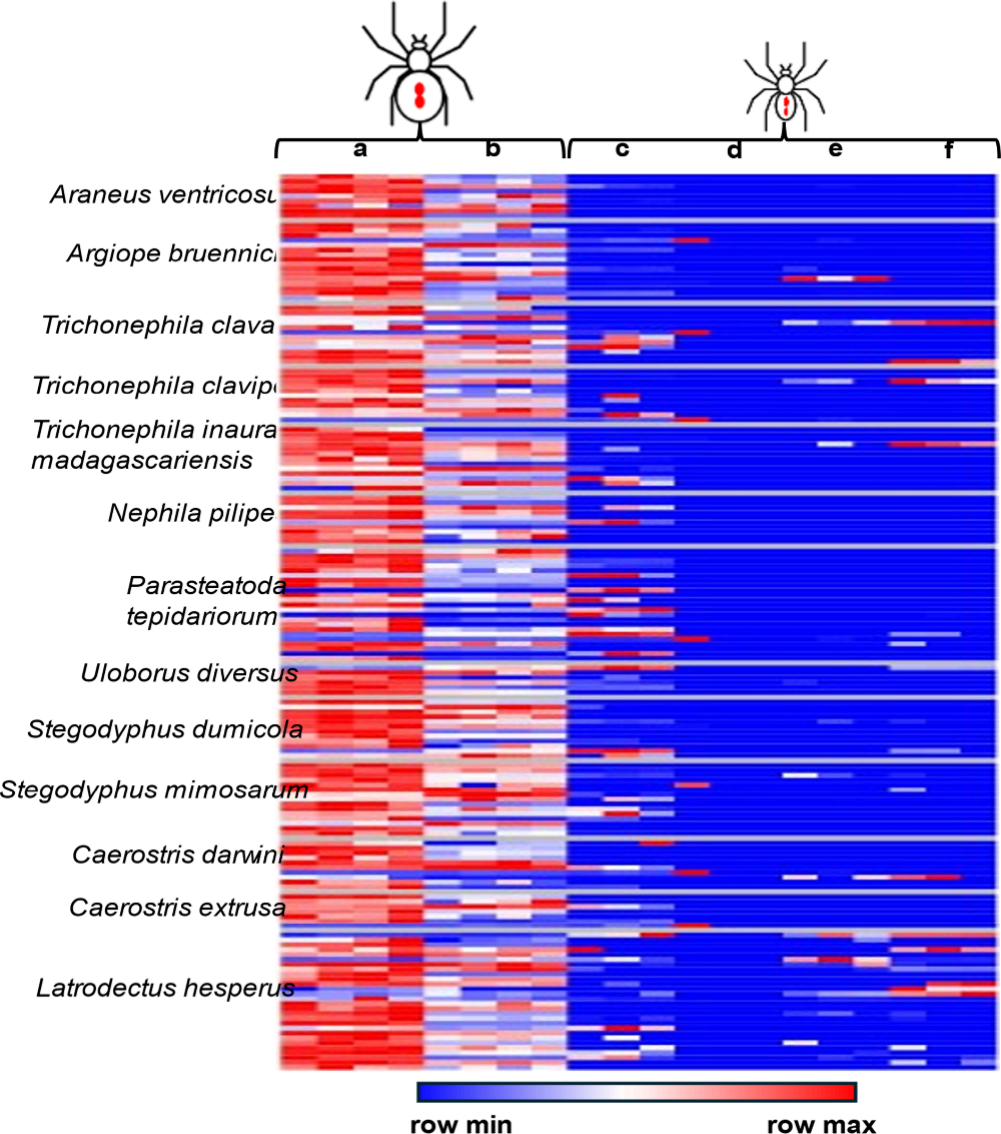
Heatmap demonstrating the relative abundance of proteins annotated as toxins in each FASTA database for each spider region analyzed. Mature female (a) body and (b) legs. Spiderling (c) abdomen, (d) head, (e) legs, and (f) sternum.

Though these results are exploratory and are more for demonstration, they do emphasize that even without the species-specific spider genome, we would have been able to make putative biological conclusions. In either case, these results would require additional work to confirm, but undoubtedly, they are hypothesis generating.

## Limitations

The 14 nonblack widow spider annotated genomes are a combination of four official RefSeq annotations and 10 user submitted annotations. This difference in annotation origin can complicate cross-species comparisons or analysis. Also, because we did not go through the ortholog mapping of over 5000 black widow proteins, there are undoubtedly duplicate protein hits or missing identifications since the FASTA used likely was not only canonical/nonredundant gene products and contained putative assignments and fragmented sequences. We expect that in the future when RefSeq releases the black widow genome annotation using this same genome assembly, the protein identifications will be more useful and this search and compilation can be reperformed. Still, this does not negate the findings and comparisons here, especially as our goal was not to drive a new biological insight but instead to provide a draft resource upon which to improve in the future. Finally, as pointed out during peer review, it can be particularly challenging to accurately estimate false discovery rates from peptide centric data-independent analysis studies without a ground truth and validation data set. As these are difficult to validate even in the most well-annotated organisms, one suggested analysis method was to see to what level peptide and protein matches would occur if we used nonspider databases. When repeating the analysis of all spider LCMS data files against the well annotated Human.fasta on hand, we matched 1445 total precursors, leading to the identification of 498 protein groups. An analysis of the well-characterized model plant *A. thaliana* resulted in 193 precursor matches in total leading to the assignment of 93 protein groups. When employing the well annotated FASTA for the American cockroach, identifications were closer to those of human, with 2067 precursors resulting in 576 protein groups. While imperfect, it does appear that throwing any protein sequences into this pipeline will not result in similar numbers of identifications as found in spider-specific databases and increases our confidence in these results.

## Conclusions

There is no question that this is an exciting time to be doing proteomics.^24^ The field may even be emerging from under the shadow of genomics and receiving mainstream attention thanks to recent success stories in the clinic and new data sets featuring thousands or tens of thousands of patient proteomes.^6^ New reagents and sample preparation methods even allow the proteomic analysis of body fluids with extremely high dynamic ranges in protein concentration, which have traditionally been the hardest things to study with proteomics.^4^ It seems no coincidence that most stories to reach the mainstream press have been in human proteomics, which is supported by the availability of both small curated genomes and vast libraries of human mutations.^25^ New resources now attempt to address population level genetic variations to enable comprehensive studies of people with racial and ethnic backgrounds under-represented in current databases.^26^ When you step outside of human studies, however, many of these resources have no parallel and seem to suggest that you simply can not do proteomics outside of a few model organisms. However, it should be noted that thousands of FASTA databases are available for analysis today and that they can be readily created by bioinformaticians from the genomic data that has been acquired or is being output on sequencers today.

Even in the absence of species-specific FASTA databases, there are many reasons to perform proteomic analysis when samples are available. The first is that there is likely sufficient protein sequence homology in the FASTA files that are available to make matches. In the presented case, every spider fasta allowed the identification of over 2000 proteins, and every library correctly identified the body of the mature female spider as the one with the highest relative concentration of toxin proteins. With freely available tools for protein annotation, we were able to match over 5500 predicted protein coding regions to spectra and assign putative annotations to over 90% of these protein level matches using orthology mapping to all available Araneidae sequences on UniProtKB. The publication of the black widow genome assembly occurred by coincidence within the same time frame as our study,^18^ and the author’s annotation was graciously provided by the authors. If this species-specific genome annotation had not been published before we completed our analysis, we could have waited. While the number of organisms on earth is large, we are constantly told that genomic technology is improving in power and decreasing in relative cost per sample. We optimistically hope that eventually genomic data will be available for every organism on earth. As such, we believe someone would have inevitably sequenced the genome of a black widow spider. If we wait a bit longer, it is likely that a fully annotated reference FASTA for this famously toxic spider will become available for an even more complete analysis of these publicly available proteomics files we have generated. However, while we identify far fewer proteins using nonspecies-specific databases, we find that those results can still be biologically meaningful and did not require waiting for complete annotation of all protein coding regions for the species in question. If there is a take away from this backyard proteomics study, it is that the correct time to do proteomics is whenever you have protein to analyze. Though it may seem frivolous to engage scientific curiosity in the world around us, there is a legitimate need for data from a broader diversity of species beyond model organisms. State-of-the-art algorithms of peptide spectra prediction and de novo are using public data for training and validation, with nearly half of public data sets on PRIDE being human derived.^12,27^ While obviously limited in scope, this case study suggests that the tools trained on human peptides can be applied effectively to less studied organisms. Together, we feel this study adds further support that there is value in generating data from species that are not human or even model organisms, including areas of the tree of life that are nearly completely lacking, such as insects and specifically spiders. Every gap found in the applications of proteomic technology to new organisms may ultimately be extremely useful in further training, in validation, or in benchmarking algorithms.

## Supplementary Material









## Data Availability

Peptide level analyses and all.fasta files exceed the maximum allowable file size for this journal and have been permanently published on FigShare and are available at the following 10.6084/m9.figshare.28462295.v1. All raw and processed proteomic data are available through the ProteomeXchange repository as accession PXD051601.

## Notes

The original CDS protein level matches in sheet 1 as well as the assigned annotations and annotation scoring parameters combined with the protein level report as sheet 2 (XLSX)
Summary table as well as individual sheets for all quantitative protein values for all samples against each respective.fasta file (XLSX)
Each toxin protein identified when using each respective.fasta file along with the quantitative source data used to generate Figure 2 (XLSX)
Supplemental Figures S1–S6­(PDF)
